# Pituitary and adrenal involvement in diffuse large B-cell lymphoma, with recovery of their function after chemotherapy

**DOI:** 10.1186/1472-6823-13-45

**Published:** 2013-10-09

**Authors:** Yasuhiro Nakashima, Motoaki Shiratsuchi, Ichiro Abe, Yayoi Matsuda, Noriyuki Miyata, Hirofumi Ohno, Motohiko Ikeda, Takamitsu Matsushima, Masatoshi Nomura, Ryoichi Takayanagi

**Affiliations:** 1 Department of Medicine and Bioregulatory Science, Graduate School of Medical Sciences, Kyushu University, 3-1-1 Maidashi, Higashi-ku, Fukuoka 812-8582, Japan

**Keywords:** Pituitary lymphoma, Adrenal lymphoma, Diffuse large B-cell lymphoma, Panhypopituitarism, Autologous hematopoietic stem cell transplantation, Recovery of pituitary and adrenal function

## Abstract

**Background:**

Diffuse large B-cell lymphoma sometimes involves the endocrine organs, but involvement of both the pituitary and adrenal glands is extremely rare. Involvement of these structures can lead to hypopituitarism and adrenal insufficiency, and subsequent recovery of their function is rarely seen. The present report describes an extremely rare case of pituitary and adrenal diffuse large B-cell lymphoma presenting with hypopituitarism and adrenal insufficiency with subsequent recovery of pituitary and adrenal function after successful treatment of the lymphoma.

**Case presentation:**

A 63-year-old Japanese man was referred to our hospital due to miosis, ptosis, hypohidrosis of his left face, polydipsia and polyuria. ^18^F-fluorodeoxy glucose positron emission tomography / computed tomography revealed hotspots in the pituitary gland, bilateral adrenal glands and the apex of his left lung. Surgical biopsy from the pituitary lesion confirmed the diagnosis of diffuse large B-cell lymphoma, with lymphoma cells replacing normal pituitary tissue. Endocrine function tests revealed adrenal insufficiency and panhypopituitarism, including a possible affection of the posterior pituitary. Hormone replacement therapy with desmopressin and hydrocortisone was started. Chemotherapy consisted of six courses of R-CHOP (rituximab, cyclophosphamide, vincristine, doxorubicin and prednisolone) and two courses of high-dose methotrexate followed by autologous hematopoietic stem cell transplantation. Subsequently, his pituitary and bilateral adrenal lesions resolved, and serial endocrine function tests showed gradual improvement in pituitary and adrenal function.

**Conclusions:**

The present report describes an extremely rare case of diffuse large B-cell lymphoma with involvement of both the pituitary and bilateral adrenal glands. R-CHOP and high-dose methotrexate therapy followed by autologous hematopoietic stem cell transplantation was quite effective, and panhypopituitarism and adrenal insufficiency improved to almost normal values after successful treatment of the lymphoma with chemotherapy.

## Background

Diffuse large B-cell lymphoma (DLBCL) sometimes involves the endocrine organs, but the involvement of both the adrenal and pituitary glands is extremely rare, both at the time of initial presentation and late in the disease course [1,2]. Similar to pituitary adenomas, pituitary lymphomas may present with symptoms of anterior pituitary hormone dysfunction. In most cases with pituitary involvement of lymphoma, hormonal dysfunction is irreversible [3,4]. The present report describes a rare case of panhypopituitarism and diabetes insipidus due to the involvement the pituitary gland and the adrenal glands with lymphoma. However, pituitary and adrenal function was restored after successful chemotherapy and autologous hematopoietic stem cell transplantation.

## Case presentation

**Figure 1:**
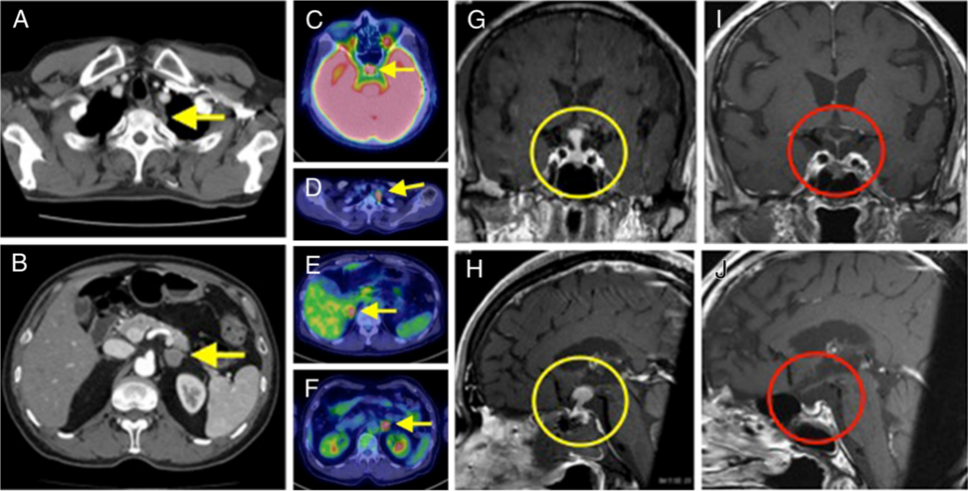
**Imaging analysis. A**. Chest computed tomography (CT) shows lesion at the apex of the left lung (yellow arrow). **B**. Abdominal CT shows a left adrenal lesion (yellow arrow). **C**, **D**, **E**, **F**. ^18^F-fluorodeoxy glucose positron emission tomography (^18^F-FDG PET) / CT. Arrows indicate high uptake in the pituitary gland **(C)**, left lung lesion **(D)**, and bilateral adrenal glands (**E** and **F**). **G** and **H**. Pre-treatment pituitary T1-weighted magnetic resonance imaging (MRI). **G**. Coronal section. **H**. Sagittal section. Suprasellar lesion (φ13 mm) and thickening of the pituitary stalk (circled in yellow). **I** and **J**. Post-treatment pituitary T1-weighted magnetic resonance imaging (MRI). **I**. Coronal section. **J**. Sagittal section. After chemotherapy, pituitary lesion disappeared (circled in red).

**Figure 2:**
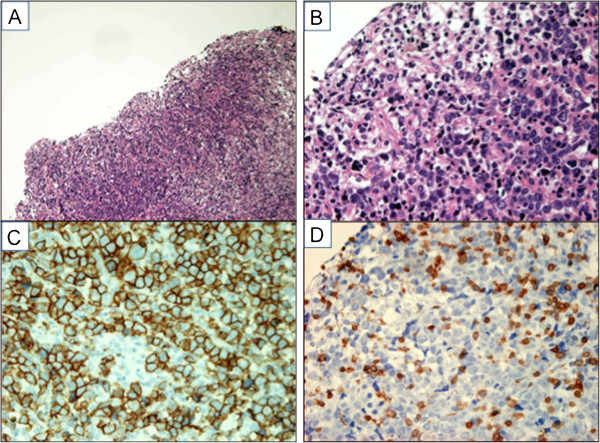
**Histology of the pituitary lesion. A**. Hematoxylin-Eosin (HE) stain (×100). The pituitary gland is completely replaced by abnormal lymphocytes. **B**. HE stain (×400). The sections show diffuse proliferation of large-sized abnormal lymphoid cells. **C**. CD20 immunostaining (×400). These atypical cells are positive for CD20. **D**. CD3 immunostaining (×400). These atypical cells are negative for CD3.

A 63-year-old man was referred to our hospital due to miosis, ptosis, hypohidrosis of his left side, polydipsia and polyuria. Computed tomography (CT) showed multiple lesions involving the pituitary gland, the apex of the left lung (Figure 1A), and the left adrenal gland (Figure 1B). ^18^F-fluorodeoxy glucose positron emission tomography / CT (^18^F-FDG PET/CT) imaging indicated high ^18^F-FDG uptake in the pituitary gland, bilateral adrenal glands and the lesion at the apex of his left lung (Figure 1C-F). Magnetic resonance imaging (MRI) of the brain revealed an enhanced suprasellar lesion (φ13 mm) and thickening of the pituitary stalk (Figure 1G,H). Surgical biopsy of the pituitary lesion showed that the pituitary gland was almost completely replaced by large abnormal lymphocytes (Figure 2A,B), which were positive for B-cell markers (CD20) (Figure 2C), but negative for T-cell markers (CD3) (Figure 2D).

Laboratory data on admission are shown in Table 1. Mild anemia and slight elevation of lactate dehydrogenase were seen, and soluble interleukin-2 receptor (sIL-2R) levels were within the normal range. Bone marrow aspiration and cerebrospinal fluid examination were negative for the presence of lymphoma cells. Basal levels of growth hormone (GH), luteinizing hormone (LH), follicle stimulating hormone (FSH), plasma adrenocorticotropic hormone (ACTH), serum cortisol, thyroid stimulating hormone (TSH), free thyroxine (f-T4), testosterone, dehydroepiandrosterone (DHEA) and aldosterone were low, and endocrine function tests revealed hypo-responsiveness to stimulation, indicating panhypopituitarism and adrenal insufficiency (Table 2). As the patient had polydipsia and polyuria (intake and urine volume approximately 5 liters / 24 hours)) with excessive thirst, and further testing showed low urinary osmolality and low antidiuretic hormone compared with serum osmolality (Table 1), the diagnosis of diabetes insipidus could not be excluded. The overall condition of the patient did not allow a deprivation test.

**Table 1:** Laboratory data on admission

**CBC**			**Serum chemistry**			**Tumor marker**				**Urinary data**		
WBC	5960	/μl	TP	6.1	g/dl	sIL-2R	702.3	(206–713)*	U/ml	SG	1.002	
Neut	53.4	%	Alb	3.8	g/dl	CYFRA	2	(< 3.5)*	ng/ml	Osmolality	178	mOsm/kg/H_2_O
Lym	36.9	%	BUN	14	mg/dl	ProGRP	49.9	(< 81)*	pg/ml	Na	18	mmol/l
Mo	8.2	%	Cre	0.88	mg/dl	CEA	1.3	(< 5.0)*	ng/ml	Volume	5475	ml/day
Eo	1.2	%	UA	3.9	mg/dl							
Ba	0.3	%	T-bil	0.8	mg/dl	**Endocrinology (basal hormone levels)**						
RBC	363×104	/μl	D-bil	0.4	mg/dl	GH	0.4	(< 2.47)*	ng/ml			
Hb	11.9	g/dl	AST	13	U/l	PRL	20.9	(4.29-13.69)*	ng/ml			
Plt	18.4×104	/μl	ALT	16	U/l	LH	0.2	(0.79-5.72)*	mU/ml			
			ALP	175	U/l	FSH	1	(2.00-8.30)*	mU/ml			
**Coagulation**			γ-GTP	20	U/l	ACTH	2	(7.2-63.3)*	pg/ml			
PT %	111	%	LDH	230	U/l	Cortisol	2.4	(4.0-18.3)*	mg/dl			
APTT	29.9	sec	Na	139	mmol/l	IGF-1	105	(75–226)*	ng/ml			
Fib	255	mg/dl	K	3.8	mmol/l	TSH	0.04	(0.5-5.0)*	μU/ml			
D-Dimer	0.9	μg/ml	Cl	100	mmol/l	f-T4	0.86	(0.90-1.70)*	ng/dl			
			Ca	8.7	mg/dl	ADH	0.5	(0.3-3.5)*	pg/ml			
**Infection**			CK	19	U/l	Testosterone	< 0.02	(1.31-8.71)*	ng/ml			
QFT	(+)		CRP	0.06	mg/dl	DHEA-EIA	7	(24–244)*	μg/dl			
			Osmolality	288	mOsm/kg/H_2_O	Aldosterone	11.4	(35.7-240)*	pg/ml			
						Renin activity	0.7	(0.3-2.9)*	ng/ml/hr			

*reference data.

*QFT* QuantiFERON TB2G.

*SG* specific gravity.

Pathologic parameters were underlined.

**Table 2:** Results of endocrine function tests on admission

**CRH/TRH/LHRH loading test**						
Min.	0	15	30	60	90	120
LH (mU/ml)	<0.2	<0.2	<0.2	<0.2	<0.2	<0.2
FSH (mU/ml)	<1.0	1.1	1.8	2.5	3.1	3.5
PRL (ng/ml)	20.9	39.8	38.7	29.8	26.4	25.9
TSH (μU/ml)	0.04	0.12	0.17	0.2	0.21	0.19
ACTH (pg/ml)	<2.0	5.8	8.9	7.8	5.7	4.8
Cortisol (μg/ml)	2.4	2.3	2.2	2	1.8	4.8
GHRP-2 loading test						
Min.	0	15	30	45	60	
GH (ng/ml)	0.4	2.3	2.5	1.7	1.1	
ACTH loading test						
Min.	0	30	60	120		
Cortisol (μg/ml)	2	3.8	4.6	5		

**Figure 3:**
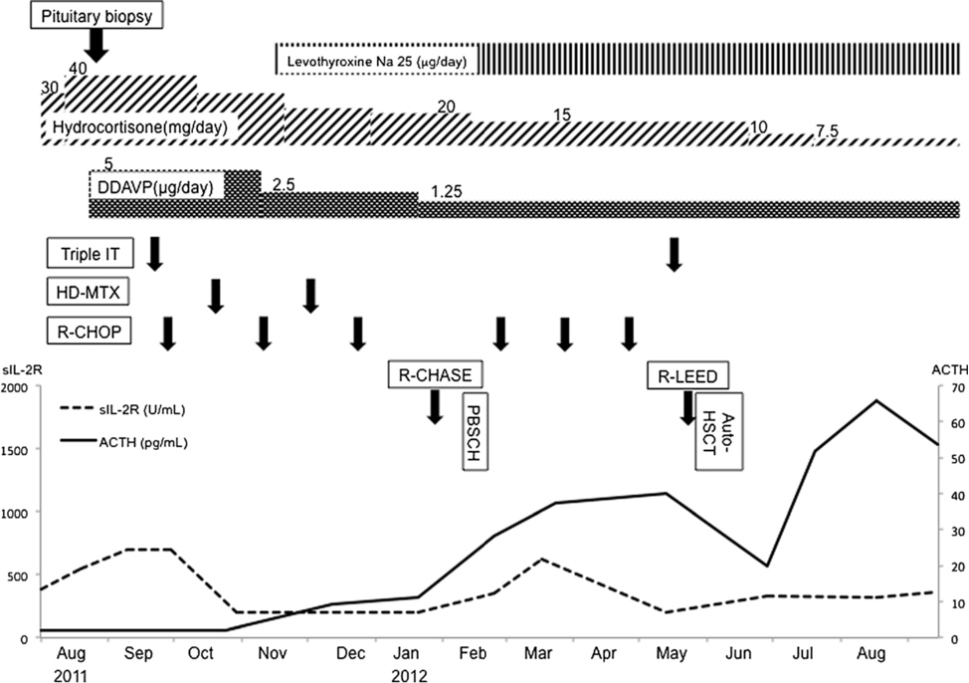
**Clinical course.** Hormone replacement therapy for pituitary and adrenal insufficiency, and chemotherapy for DLBCL were performed. Line graphs show the transition of soluble interleukin-2 receptor (sIL-2R) and basal levels of ACTH.

Based on these data, the patient was diagnosed with DLBCL with pituitary and adrenal involvement. Hormone replacement therapy was initiated with thyroxine (25 μg/day), desmopressin (5 μg/day), and hydrocortisone (30–40 mg/day). Since his international prognostic index was consistent with poor prognosis, we performed six cycles of chemotherapy with rituximab (375 mg/m^2^, day 1), cyclophosphamide (750 mg/m^2^, day 2), vincristine (1.4 mg/m^2^, max 2 mg/body, day 2), doxorubicin (50 mg/m^2^, day 2) and prednisolone (100 mg/body/day, day 1–5) (R-CHOP) followed by planned autologous hematopoietic stem cell transplantation (auto-HSCT). To prevent infiltration of DLBCL into the central nervous system, two cycles of intravenous high-dose methotrexate (3.5 g/m^2^/day, day 1) therapy (HD-MTX) and two cycles of intrathecal injection of MTX (15 mg/body), cytarabine (40 mg/body), and dexamethasone (3.3 mg/body) were performed (Figure 3). Following administration of these chemotherapies, the pituitary and bilateral adrenal lesions disappeared on MRI, CT, and ^18^F-FDG PET/CT. Although the lung lesion remained on CT scan, no significant ^18^F-FDG uptake was shown in PET/CT, so the lung lesion was considered as not viable. The level of sIL-2R had been within reference range during the chemotherapy (Figure 3).

**Figure 4:**
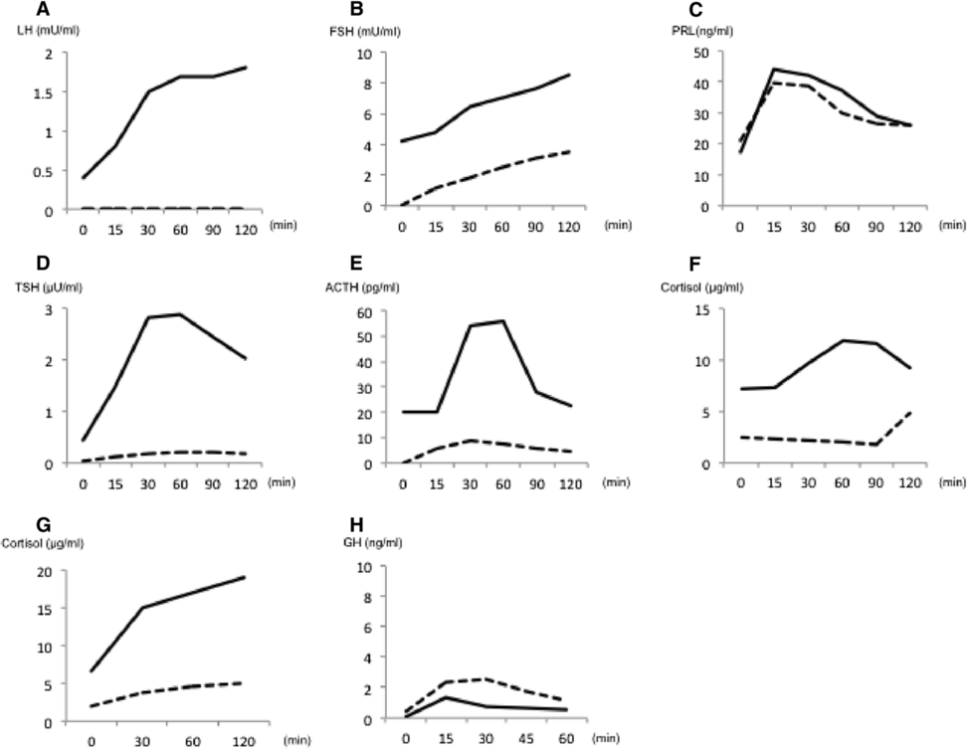
**The results of endocrine function tests before and after chemotherapy. (A, B)** The response of LH **(A)** and FSH **(B)** to LHRH loading test. **(C, D)** The response of PRL **(C)** and TSH **(D)** to TRH loading test. **(E, F)** The response of ACTH **(E)** and cortisol **(F)** to CRH loading test. **(G)** The response of cortisol to ACTH loading test. **(H)** The response of GH to GHRP-2 loading test. Dashed lines show the data of each hormone at diagnosis, and solid lines show those after chemotherapy.

The results of endocrine function tests before and after chemotherapy are shown in Figure 4. Panels A-F show the results of corticotropin-releasing hormone (CRH), thyrotropin-releasing hormone (TRH), and the LH-releasing hormone (LHRH) loading test. These 3 loading tests were combined and performed at the same time. Secretion of LH, FSH, TSH, and ACTH were severely suppressed at the time of diagnosis, but dramatically improved after the completion of chemotherapy (Figure 4A,B,D,E), and the basal ACTH level improved to the upper limit of the reference range, according to the reduction of hydrocortisone.

Although the basal and stimulated values of PRL were within the reference range before treatment, a decrease in the basal level and an increase in the peak level were found after successful treatment of the lymphoma, suggesting resolution of hypothalamic compression (Figure 4C). This notion was supported by results for head MRI, showing disappearance of the suprasellar region projecting to the hypothalamus after treatment of the lymphoma (Figure 1I and J). As shown in Figure 4F and 4G, the impairment of cortisol secretion was explained by the functional defects in the pituitary gland. After chemotherapy, improvement in cortisol secretion indicated recovery from secondary hypoadrenalism. Considering the involvement of DLBCL in bilateral adrenal glands, a possibility of primary hypoadrenalism cannot be excluded. Nonetheless, the successful treatments lead to the recovery of adrenal insufficiency. Figure 4H shows the results of the GH releasing protein-2 (GHRP-2) loading test; the deficit in secretion of GH persisted after chemotherapy. As the treatment progressed, the symptoms of diabetes insipidus decreased, and the desmopressin dose was gradually decreased to 1.25 μg/day, and then, the requirement of hydrocortisone was also decreased to 7.5 mg/day (Figure 3). Under this replacement regimen, basal hormone levels of cortisol, TSH and f-T4 improved to 15.8 μg/dl, 0.43 μU/ml and 0.94 ng/dl respectively. The basal level of testosterone was not improved (0.02 ng/ml). The basal level of testosterone was not improved, so testosterone replacement therapy will be initiated with the next follow-up visits.

## Discussion

The present report describes an extremely rare case of DLBCL with pituitary and bilateral adrenal involvement. There are only three such cases reported in the literature (Table 3), all of which involved elderly males. Two cases involved DLBCL, while the remaining case involved diffuse large cell non-Hodgkin’s lymphoma, but immunohistochemical data were not available. Frequent primary symptoms in those cases were ptosis caused by oculomotor nerve infiltration or compression, hyponatremia, and weakness due to corticosteroid insufficiency. Other involved sites of the lymphoma that varied with the specific case. Among primary symptoms in our case, miosis, ptosis, and hypohidrosis of his left face were similar to a pancoast tumor. Since the lesion in the apex of the left lung persisted on CT, but its ^18^F-FDG uptake in ^18^F-FDG PET/CT disappeared after intensive chemotherapy, we consider that this lesion was also a nidus of malignant lymphoma. Furthermore, polydipsia and polyuria suggested the existence of pituitary lesion, and several imaging examinations, especially ^18^F-FDG PET/CT, were useful to evaluate the localization of the lesions. For histological confirmation, we examined a pituitary lesion by Hardy’s operation, which was considered to be the operative strategy associated with the lowest burden on the patient. Finally, a successful diagnosis of DLBCL was made, which is the most common diagnosis among those with pituitary and adrenal lymphomas (Table 3).

**Table 3:** Summary of case reports with pituitary and adrenal involvement in patients with lymphoma

**Age/gender**	**Pathology**	**Presenting symptoms**	**Involved sites**	**Lymphoma treatment**	**Hormonal treatment**	**Prognosis, survival**	**Reference**
77/M	NHL diffuse large cell	Hyponatremia, Hypoglycemia, Weakness, Confusion	Pituitary gland, Bilateral adrenal glands	No treatment	T4, GC	Died, 9 weeks	[5]
59/M	DLBCL	Weakness, Ptosis, Mild Hyponatremia, Headache	Pituitary gland, Bilateral adrenal glands	R-CHOP, IT MTX	T4, GC, T, Fludro	Alive, 18 months	[6]
77/M	DLBCL	Fever, Hyponatremia, Ptosis	Pituitary gland, Bilateral adrenal glands, Liver, Spleen, Bone marrow	CHOP, IT MTX	GC	Died, 12 months in remission	[1]
63/M	DLBCL	Polyuria, Polydipsia, Miosis, Ptosis, Hypohidrosis of his left side	Pituitary gland, Bilateral adrenal glands, Lung	R-CHOP, HD-MTX, IT MTX, auto-HSCT	T4, GC, Desmo	Alive, 15 months	This report

*M* male, *NHL* non-Hodgkin’s lymphoma, *DLBCL* diffuse large B-cell lymphoma, *CHOP* cyclophosphamide, doxorubicin, vincristine, prednisolone, *IT* intrathecal injection, *MTX* methotrexate, *R-CHOP* rituximab combined with CHOP.

*HD* high dose, *auto-HSCT* autologous hematopoietic stem cell transplantation, *T4* thyroxin, *GC* glucocorticoid, *T* testosterone, *Desmo* desmopressin, *Fludro* fludrocortisone.

Although histological examination was performed only in the pituitary lesion, the efficacy of chemotherapy in terms of resolving the adrenal and lung lesions suggests that those lesions were also malignant lymphoma.

Except for one patient who did not receive chemotherapy, CHOP or R-CHOP chemotherapy led to sustained remission (Table 3). To address concerns regarding the risk of infiltration of lymphoma cells into the cerebrospinal fluid, intrathecal injection of MTX was performed in three cases, and no central nervous system relapse was reported. In the present case, autologous peripheral stem cell transplantation was performed in addition to chemotherapy. Non-Hodgkin’s lymphoma can be classified into four different groups according to international prognostic index, and worse prognosis is associated with the high-intermediate- and high-risk groups [7]. Auto-HSCT has been tried to improve survival of the high-risk cases.

In the present case, pituitary and adrenal function was severely impaired at the time of initial diagnosis. However, as the lesions reduced in response to chemotherapy, pituitary and adrenal function gradually improved, allowing a reduction in the dose of hormone replacement therapy. Some case reports of pituitary lymphoma have described the restoration of basal hormone levels after successful chemotherapy [8,9]; the precise mechanism of recovery in those cases was not clear, but small unaffected tissues might compensate for the pituitary and adrenal insufficiency. To avoid excess hormonal replacement, follow-up evaluation of endocrine function during and after chemotherapy is highly recommended.

## Conclusion

In conclusion, the present study described an extremely rare case of malignant lymphoma with involvement of the pituitary and bilateral adrenal glands. Combined therapy with CHOP and HD-MTX followed by auto-HSCT was quite effective, and panhypopituitarism and adrenal insufficiency improved to almost normal values after treatment of the lymphoma with chemotherapy. Although pituitary and adrenal lymphomas are rare, it is important to diagnose lymphomatous infiltration to those structures in the early stage of the disease, because pituitary and adrenal dysfunction might be reversible. Careful endocrinological follow up is also needed to assure appropriate adjustments in dosages of hormone replacement therapy in patients who might have changes in endocrine function for successful treatment of the invading neoplasm.

## Consent

Written informed consent was obtained from the patient for publication of this Case report and any accompanying images. A copy of the written consent is available for review by the Editor of this journal.

## Abbreviations

DDAVP: 1-desamino-8-D-arginine vasopressin; R-CHASE: Rituximab (375 mg/m2, day 1), cyclophosphamide (1,200 mg/m^2^, day 2), cytarabine (2 g/m^2^/day, day 2–3), etoposide (100 mg/m^2^/day, day 1–3), dexamethasone (33 mg/body/day, day 1–3); R-LEED: Rituximab (375 mg/m^2^, day 1), etoposide (500 mg/m^2^/day, day 2–4), cyclophosphamide (60 mg/kg/day, day 3–4), melphalan (130 mg/m^2^/day, day 5), dexamethasone (33 mg/body/day, day 2–5); PBSCH: Peripheral blood stem cell harvest; Auto-HSCT: Autologous hematopoietic stem cell transplantation.

## Competing interests

The authors declare that they have no competing interests.

## Authors’ contributions

YN designed and drafted the manuscript and interpreted data. MS, MN and RT revised the manuscript. IA, YM and NM participated in the endocrinological treatment, and collected the data. YN, HO, MI, TM and MS participated in the hematological treatment of the patient. All authors read and approved the final manuscript.

## Pre-publication history

The pre-publication history for this paper can be accessed here:

http://www.biomedcentral.com/1472-6823/13/45/prepub
